# Acquired cancer tyrosine kinase inhibitor resistance: ROS as critical determinants

**DOI:** 10.1038/s41392-021-00844-5

**Published:** 2021-12-24

**Authors:** Sander Bekeschus

**Affiliations:** ZIK plasmatis, Leibniz Institute for Plasma Science and Technology (INP), Felix-Hausdorff-Str. 2, 17489 Greifswald, Germany

**Keywords:** Lung cancer, Cell biology

The ability of a minority of cancer cells lineages to withstand drug-induced toxicity and re-enter cell cycling in the presence of the drug has been observed for long, but the underlying mechanisms remained elusive. New work published in *Nature* by Oren and colleagues used barcode lentiviral library labeling to prominently discern antioxidant profiles, among others, being the primary underlying cellular program that governs proliferation under tyrosine kinase inhibitor (TKI) drug pressure.^1^

**Fig. 1:**
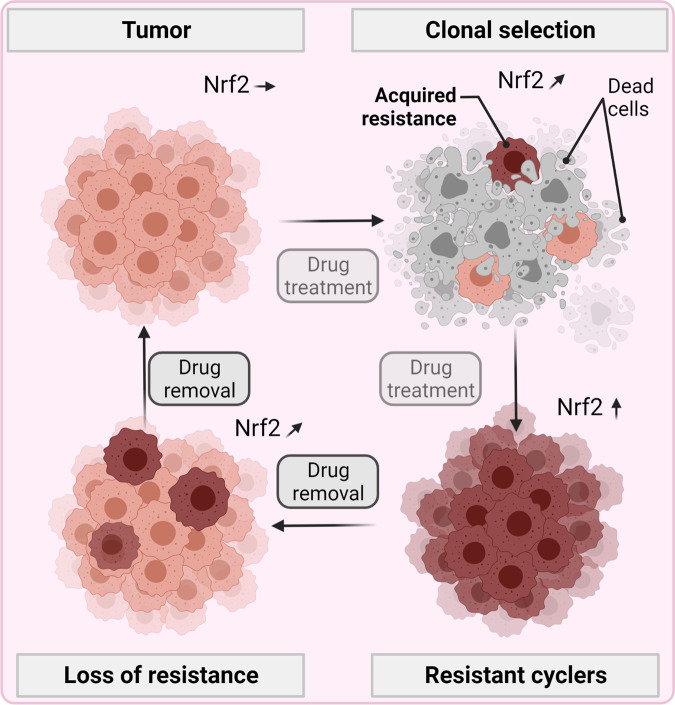
Cycling persisters upon TKI drug pressure. In unchallenged tumor cells with physiological Nrf2 levels, drug exposure leads to a clonal selection of cells with acquired drug resistance characterized by supraphysiological Nrf2 levels. Only cells with high antioxidant defense levels can proliferation while drug-resistant cells with lower levels do not. Nrf2 and antioxidant defense levels ceased after drug removal, ultimately resembling tumor cells before the initial drug challenge. Hence, drug sensitivity and resistance are dynamic cellular states controlled by a set of antioxidant defense response genes rather than separate entities a priori. Created with BioRender.com

In the study, lung cancer cells were pulsed with osimertinib, a third-generation EGFR TKI. After two weeks, two main rare populations were identified: non-cycling persisters that survived drug treatment and cycling persisters proliferating under drug burden. The elegance of the experiments lies in the utilization of a lentiviral barcode library called Watermelon, allowing back-tracing and transcriptional profiling of the initial progenitor cells present before drug addition that gave rise to the surviving cells two weeks later.^1^ Thus, this study design is fundamentally different from most reports investigating the eventually-arising drug-resistant cells rather than their constituting progeny (Fig. 1).

Cycling persisters are responsible for tumor relapse in patients. The overarching hallmark of this cell population was a redox profile shifted towards antioxidant responses, as characterized by increased glutathione metabolism and Nrf2 gene signatures and decreased ROS levels. This was seen in several cell lines in vitro, minimum residual disease lung tumors in vivo, and as collective transcriptional ROS signature in residual disease lung, melanoma, and breast tumor samples derived from patients after oncogenic kinase inhibitor treatment. In vitro, through the Watermelon system, these signatures were found to be induced through drug challenge in distinct lineages rather than being present a priori. The role of ROS and antioxidants was validated as the percentage of cycling persisters was increased by genetic suppression of the Nrf2 negative regulator Keap1, overexpression of the transcriptional Nrf2 target GPX2, and to the greatest extent by the addition of the antioxidant NAC. By contrast, a decline was observed with pharmacological inhibition of the cysteine-glutamate antiporter xCT crucial for synthesizing the antioxidant glutathione. Strikingly, non-cycling persisters did not show such (but instead other) signatures, suggesting ROS control to be the decisive element governing the fate of tumor cells not only in terms of drug resistance but also in retaining proliferate capacity.

These results do not seem overly surprising, considering the many existing reports on Nrf2 and antioxidant defense in drug resistance. At the same time, the study is novel in the way that it shows that the inducibility of the Nrf2 pathway is critical for cell survival under drug pressure rather than its preexisting activity on a single cell level rather than bulk profiling. Moreover, cycling persisters under drug pressure were re-seeded in the absence of drugs, giving rise to progeny with similar drug sensitivity profiles as found initially, ruling out genetic mechanisms causing drug resistance in this study. However, the underlying question is where the ROS are coming from that elicit the Nrf2 pathway in the first place. The EGFR targeting of osimertinib deprives lung cancer cells of pro-survival signaling, and ROS are known to be generated during pro-apoptotic processes and autophagy. As ROS, Nrf2, and antioxidant signatures were the main hallmark of cycling persisters in this study, it can be assumed that these are decisive for drug resistance and retained proliferation. A key study supporting these findings was made by Yamadori and colleagues published in 2012 in *Oncogene* using PC-9 cells, among others, as in the *Nature* study.^2^ They reported that EGFR-dependent proliferation in PC-9 cells could be circumvented when inducing oxidative stress generated through cigarette smoke, which inactivated Keap1 to free Nrf2, eventually unleashing its three-fold battery of cellular defense mechanism, namely antioxidant genes, phase II detoxifying enzymes, and drug efflux pumps. The results were confirmed in A549 cells harboring constitutively active Nrf2 signaling due to a mutation in Keap1, rendering the cells intrinsically insensitive to TKI-induced proliferation suppression.

This proliferation seen under TK inhibition with concomitant Nrf2 activation prompts two key questions. First, where is the ROS coming from in the *Nature* study to spur NRf2 activity? Second, why is such a mechanism only active in the small subset of cycling persisters but not all cells? The cellular dynamics of the second question remain to be unraveled, as the *Nature* study showed that drug-resistant cyclers’ transcriptional profile did not significantly differ from other cells before drug addition and that they regained drug-sensitivity during prolonged culture in the absence of TKI. Regarding the first question, several ROS sources are known to contribute to altering intracellular redox states, such as NADPH oxidases, dual oxidases, mitochondrial superoxide leaking from the respiratory chain activity, and reduced antioxidant activity. Moreover, excess ROS can also stem from extracellular sources, such as dyeing cells, releasing hydrogen peroxide into the microenvironment that can enter live cells through aquaporins. The ROS levels, however, must be low enough to serve as signaling molecules (oxidative eustress)^3^ to activate the Nrf2 pathway that then serves as an EGFR-independent driver of proliferation. Apart from where the initial ROS are coming from, it is unclear how the ROS levels are sustained to promote continuous Nrf2 activation. As TKI-resistance was retained only presence of osimertinib, it is conceivable that the substance may have off-target effects on the activity of ROS-generating enzymes or other proteins, such as endogenous uncouplers of the mitochondrial respiratory chain to promote ROS leakage.

Over the last years, the plot thickened on the critical roles of spatiotemporal ROS level changes and modulation in their removal capacity in cancer drug resistance. At the same time, the omnipresence of ROS and technical hurdles in their identification and quantification leads to an often underestimated role of ROS in setting pathways in motion. Thus, it is time to call for a dedicated field of Redox Oncology focusing on the roles of ROS and their regulation in preclinical and clinical cancer biology and medicine.

Finally, the question arises of how ROS can be actively engaged and locally generated to promote tumor cell demise in concert with standard therapies. Besides classical photodynamic therapy, new approaches continuously arise from the field of nanomedicine and functionalized nanomaterials. For instance, surface-oxidized nanosheets were recently fabricated and provided both ROS-dependent antitumor efficacy and fluorescence-guided imaging in preclinical cancer models as a novel theranostic platform.^4^ Another innovative local ROS-generating treatment modality is gas plasma technology, with first cancer patients benefiting during early clinical trials.^5^ However, consensus-based broader clinical application and therapy approval of such approaches is still awaited.
